# Environmentally Friendly Quinolones Design for a Two-Way Choice between Biotoxicity and Genotoxicity through Double-Activity 3D-QSAR Model Coupled with the Variation Weighting Method

**DOI:** 10.3390/ijerph17249398

**Published:** 2020-12-15

**Authors:** Peixuan Sun, Yuanyuan Zhao, Luze Yang, Zhixing Ren, Wenjin Zhao

**Affiliations:** 1 College of New Energy and Environment, Jilin University, Changchun 130012, China; sunpx19@mails.jlu.edu.cn (P.S.); yanglz19@mails.jlu.edu.cn (L.Y.); 2 College of Environmental Science and Engineering, North China Electric Power University, Beijing 102206, China; zyy0210@ncepu.edu.cn; 3 College of Forestry, Northeast Forestry University, Harbin 150040, China; renzhixingryy@outlook.com

**Keywords:** quinolones, ryegrass, biotoxicity, molecular docking, 3D-QSAR, combined toxic effects of antibiotics, molecular dynamics

## Abstract

Quinolone (QN) antibiotics are widely used, which lead to their accumulation in soil and toxic effects on ryegrass in pasture. In this study, we employed ryegrass as the research object and selected the total scores of 29 QN molecules docked with two resistant enzyme structures, superoxide dismutase (SOD, PDB ID: 1B06) and proline (Pro, PPEP-2, PDB ID: 6FPC), as dependent variables. The structural parameters of QNs were used as independent variables to construct a QN double-activity 3D-QSAR model for determining the biotoxicity on ryegrass by employing the variation weighting method. This model was constructed to determine modification sites and groups for designing QNs molecules. According to the 3D contour map of the model, by considering enrofloxacin (ENR) and sparfloxacin (SPA) as examples, 23 QN derivatives with low biotoxicity were designed, respectively. The functional properties and environmental friendliness of the QN derivatives were predicted through a two-way selection between biotoxicity and genotoxicity before and after modification; four environmentally friendly derivatives with low biotoxicity and high genotoxicity were screened out. Mixed toxicity index and molecular dynamics methods were used to verify the combined toxicity mechanism of QNs on ryegrass before and after modification. By simulating the combined pollution of ENR and its derivatives in different soils (farmland, garden, and woodland), the types of combined toxicity were determined as partial additive and synergistic. Binding energies were calculated using molecular dynamics. The designed QN derivatives with low biotoxicity, high genotoxicity, and environmental friendliness can highly reduce the combined toxicity on ryegrass and can be used as theoretic reserves to replace QN antibiotics.

## 1. Introduction

Antibiotics are a class of active drug compounds widely used in human and veterinary medicines, aquaculture, and agriculture [1], and which are often employed to prevent or treat microbial infections [2]. Quinolone (QN) antibiotics have emerged as one of the most commonly used antibiotics worldwide because of their wide antibacterial spectrum, high efficiency, and strong bactericidal effect [3,4]. However, compared with other types of antibiotics, QNs are characterized by their long biological half-life and difficult degradation [5]. Studies have shown that 60–90% of veterinary or human QNs are excreted as urine or feces and are used as fertilizers. After entering the environment, QNs pollute the soil and water through adsorption, migration, and accumulation [6] and accumulate in plants [7], leading to the problem of varying degrees of residues in water [8,9], soil [10,11], and sediments [12,13]. Studies have shown that accumulated QNs in the environment will produce a stress effect on plants. Wang et al. [14] indicated that the residual QNs are toxic to ryegrass. Lu et al. [15] found that the high concentration of norfloxacin seriously damaged the cell structure of Scenedesmus obliquus.

Perennial ryegrass is a cold-season grass, which is an excellent lawn grass that has been most frequently introduced and cultivated globally due to its simple planting process, short growth cycle, high yield, densely developed root system, and adaptability to the low-temperature environment during winter [16]. Thomas et al. [17] found that the root system and rhizosphere microorganisms of ryegrass promote the biodegradation of hydrocarbon pollutants. Morgan et al. [18] proved that the coexistence of ryegrass with shrubs and earthworms can limit nutrient overflow and soil decay. Daliya et al. [19] compared nine different species of ryegrass and reported that ryegrass from Norway exhibits the optimal drought tolerance. All the aforementioned studies have shown that ryegrass as high-quality pasture vegetation has highly significant effects on the environment, however, the wide application of QN antibiotics in domestic livestock industry will also have adverse effects on ryegrass.

In the last twenty years, domestic and foreign scholars have studied the residual status quo, biotoxicity, and degradation of antibiotics on ryegrass and other plants. David et al. [20] reported high concentrations of ciprofloxacin antibiotics in lettuce grown in a farmland near Kumasi Hospital in Ghana. Hu et al. [21] found that the residues of typical veterinary medicines such as ofloxacin and pefloxacin were present in different tissues of celery and parsley in winter in northern China. Jin et al. [22] observed that tomatoes were more sensitive to enrofloxacin (ENR) than wheat and Chinese cabbage were. Luciana et al. [23] reported that ENR and its metabolite ciprofloxacin are toxic to plants, such as cucumbers and radishes, and can be transmitted through a food chain. All these studies have indicated that QNs can result in different degrees of toxic effects on plants and human health through the food chain. However, a study [24] found that *Ceratophyllum demersum* and *Ceratophyllum zizanioides* can effectively degrade fluoroquinolones. Tuo et al. [25] reported that ryegrass can reduce the concentrations of antibiotics and antibiotic-resistance genes in soils polluted with copper and ciprofloxacin. Ciara et al. [26] studied the effects of the application of organic fertilizers to the microbial community of perennial ryegrass through pot experiment and isolated antibiotic-resistant strains. For the widely existing problem of soil pollution caused by QNs, plant degradation technology has become one of the most commonly used and promising remediation methods due to its advantages of economy, efficiency, and environmental friendliness [27,28]. Several domestic studies have been conducted in related fields. Deng et al. [29] observed that ciprofloxacin, tetracycline, and sulfadiazine had a “low-promoting and high-inhibition” effect on ryegrass germination, and the influence of sulphadiazine was higher than that of ciprofloxacin and tetracycline. Xie et al. [30] reported that the presence of tetracycline adversely affects the biomass of perennial ryegrass and reduces phosphorus assimilation in ryegrass. Zhao et al. [31] found that the degradation and accumulation rate of oxytetracycline in ryegrass were 54.02–53.75% and 16.33–17.99%, respectively, through the pot experiment and exposure test, which affected the germination potential and chlorophyll, superoxide dismutase (SOD), and malondialdehyde (MDA) contents of ryegrass. Pei et al. [32] observed that a 15.3–28.8% degradation rate of QNs can be achieved in soil by planting ryegrass. According to Li et al. [33], tetracycline and other antibiotics can be absorbed and retained in ryegrass, and the higher the concentration, the greater the residual amount. Ryegrass has a digestion effect on antibiotics. Jin et al. [34] reported that ryegrass can absorb polycyclic aromatic hydrocarbons and that endophytic bacteria can respond to the pollution, which can lead some pollutants to degrade by themselves or in collaboration with the ryegrass enzyme system. Therefore, the study of the biotoxicity effect and QN mechanism on ryegrass is highly crucial to alleviate the current condition of antibiotic-polluted pastures and human health.

In this study, a molecular docking method was employed to dock representative QN molecules with SOD and proline (Pro), which play a role in the adverse conditions of ryegrass, by using the variation weighting method to evaluate the combined effect of the two groups of docking scores. Comprehensive values (CI) and the structural parameters of QNs were used as dependent and independent variables, respectively, to construct a QN double-activity three-dimensional (3D) quantitative structure–activity relationship (3D-QSAR) model for studying the biotoxicity on ryegrass. By using ENR and sparfloxacin (SPA), 46 QN derivatives were designed based on the 3D contour map information of the double-activity 3D-QSAR model to obtain modification sites. Moreover, 46 QN derivatives were analyzed to determine their environmental friendliness, functional properties, and aquatic ecosystem, and environmentally friendly QN derivatives with low biotoxicity, high genotoxicity, and excellent repair effects on aquatic ecosystems were screened out. Various QNs and their derivatives coexist in the environment, therefore, the purpose of this study is not only to reduce the biotoxicity of single QNs, but also to reduce the combined toxicity. Ren et al. [35] calculated the binding energy between proteins and molecules by molecular dynamics method and studied the combined toxicity of residual QNs in a water environment. Molecular dynamics was used to verify the mechanism of the combined toxicity on ryegrass, which provided theoretical reserves for the modification of novel QN derivatives and the replacement of drugs.

## 2. Materials and Methods

### 2.1. Data Source of QNs Biotoxicity on Ryegrass: Molecular Docking Method

The systems of ryegrass and other plants for resistance towards adversity stress are the antioxidant enzyme system including SOD and osmotic regulation substances represented through Pro. SOD are a class of antioxidant enzymes that are commonly found in plants and animals [36]. They can be used to eliminate the reactive oxygen species (ROS) produced through the metabolism of plants and protect cells from toxic effects under some adversity stress conditions [37,38]. Pro is a non-toxic multifunctional amino acid with low molecular weight and high solubility. It is the most important and widely distributed organic osmotic regulation substance in plants [39]. It maintains the osmotic balance of the cells and reduces damage, acting as a reactive oxygen scavenging agent in the meantime [39,40]. In this study, the Protein Data Bank (PDB) database was used to obtain the structures of the aforementioned two enzymes found in plants, namely SOD (PDB ID: 1B06) from sour sandalwood leaves [41] and cytoplasmic peptidase Pro–Pro (PPEP-2, PDB ID: 6FPC) [42].

Ren et al. [43] used molecular docking to dock fluoroquinolones with a γ-aminobutyric (GABA) receptor and cyclooxygenase (COX) and the human ether-a-go-go-related gene (hERG) potassium channel proteins and characterized the toxic side effects of fluoroquinolones with total (docking) scores. Hou et al. [44] analyzed the binding rate of plasma proteins by using the total scores of QNs and the plasma proteins and designed trivaroxacin derivatives with a low binding rate of the plasma proteins. Gu et al. [45] employed molecular docking to dock polychlorinated naphthalenes and new polychlorinated naphthalenes with oxidase for characterizing the biodegradability of polychlorinated naphthalenes by employing total scores. Therefore, in this study, Discovery Studio 4.0 (DS) software was used, and 29 commonly used QN molecules were loaded into DS software as ligand molecules. A LibDock module was used to define the selected two proteins as receptor molecules. Find Sites From Receptor Cavities under the Define module were used for obtaining sites in the receptor, which may be combined, and then for modifying and defining the binding sites. A sphere with a radius of 9 was defined at the binding site by using Define Sphere from Selection under the Define module. Using the Dock Ligands module, the Docking Preferences were User Specified and the Max Hits to Save was 10. Finally, ligand molecules were integrated into the formed binding cavity of the protein for rapid docking with the receptor protein, and the capacity of the ligand to bind with the receptor was expressed as total scores, that is, biotoxicity.

### 2.2. QNs Biotoxicity Characterization to Ryegrass-Variation Weighting Method

In this study, the two groups of total scores representing the QN biotoxicity were processed and integrated into a group of data through variation weighting, which was employed for analyzing the biotoxicity of QNs to ryegrass and constructing the QNs double-activity 3D-QSAR model of biotoxicity on ryegrass. The formula for variation weighting is as follows:

The total scores of two enzymes were used as the index system and 29 QNs molecules were evaluated to obtain a data matrix of order 2 and 29:

(1) $$X=\left[\begin{array}{cc} X_{11} & X_{12}\\ X_{21} & X_{22}\\ \cdots & X_{\mathrm{ij}}\\ X_{291} & X_{292} \end{array}\right]\;i=1,2\ldots ,29\;j=1,2$$

where $X_{\mathrm{ij}}$ is the jth total score of the ith molecule.

The average value of the jth index of 29 molecules, reflecting its average level, was calculated as follows:

(2) $$\bar{X_{j}}=\frac{1}{n}\sum_{i=1}^{n}X_{j}\;i=1,2\ldots n\;j=1,2\ldots ,m\;n=29\;m=2$$

where $X_{j}$ is the total score of the jth toxicity index.

To determine the difference between the experimental and average values, the standard deviation of the jth index was calculated to reflect the absolute variation degree of the jth index:

(3) $$S_{j}=\sqrt{\sum_{i=1}^{n}\frac{{\left(X_{j}-\bar{X_{j}}\right)}^{2}}{n}}\;i=1,2\ldots ,n\;j=1,2\ldots ,m\;n=29\;m=2$$

By considering that the standard deviation coefficient is more scientific and intuitive than the mean difference, the standard deviation coefficient of the jth index was calculated as follows:

(4) $$V_{j}=\frac{S_{j}}{\bar{X_{j}}}\times 100\%\;i=1,2\ldots ,n\;j=1,2\ldots ,m\;n=29\;m=2$$

The weight of the jth index can be obtained using the normalization:

(5) $$W_{j}=\frac{V_{j}}{\sum_{j=1}^{m}V_{j}}\;i=1,2\ldots ,n\;j=1,2\ldots ,m\;n=29\;m=2$$

The static individual index of the jth index was calculated according to the weight of each index:

(6) $$H_{ij}=\frac{X_{ij}}{X_{j}}\;i=1,2\ldots ,n\;j=1,2\ldots ,m\;n=29\;m=2$$

CI, as known as the biotoxicity values of QNs on ryegrass was calculated as follows:

(7) $$\mathrm{CI}=\sum_{j=1}^{m}H_{ij}\times W_{j}\;i=1,2\ldots ,n\;j=1,2\ldots ,m\;n=29\;m=2$$

### 2.3. Molecular Modification of QNs Derivatives: Double-Activity 3D-QSAR Model

SYBYL-X2.0 software was used for 3D-QSAR analysis. The CI values and structural parameters of the 29 QNs were used as dependent and independent variables, respectively, to construct the QN double-activity 3D-QSAR model for the biotoxicity on ryegrass [46,47]. During model construction, 11 and 4 QNs were randomly selected as the training and test set, respectively, (template molecule was present in both training and test sets) to construct the double-activity 3D-QSAR model.

**Figure 1:**
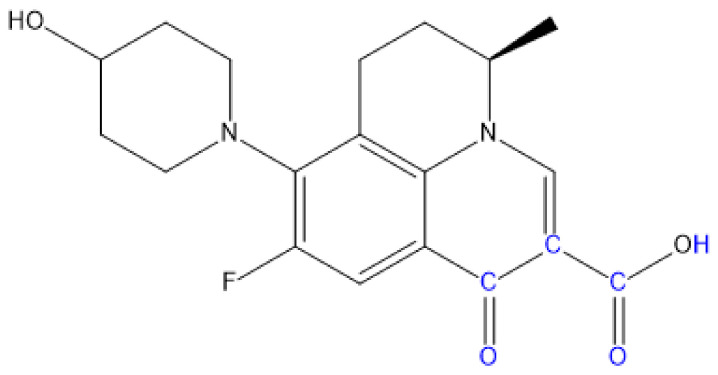
Template molecule NAD and its common skeleton.

First, 29 QNs were plotted using a sketch molecule module in SYBYL-X2.0 software. The molecular structure directly drawn in SYBYL-X2.0 software during model construction was not the most stable conformation of the molecule. For unknown receptors, the lowest energy state conformation of the molecular structure was generally selected as the active conformation. The minimized module in SYBYL-X2.0 was used for molecular optimization by employing the Powell conjugate energy gradient method. The Tripos molecular force field was selected, a Gasteiger-Huckel charge was added, and iteration was 10000. Energy convergence was limited to 0.005 kJ/mol, and other values were set to default values [48]. Moreover, the common structures of 11 QN molecules were selected as common skeletons, and the align database module was used to perform the skeleton superposition of the 11 QNs molecules. A third-generation fluoroquinolone nadifloxacin (NAD) molecule with a wide antibacterial spectrum and applications was used as the template molecule, and the blue substituents presented in Figure 1 were used as the common skeleton for superposition.

Comparative molecular field analysis (CoMFA) was conducted to analyze the relationship among compound structures, the distribution of surrounding molecular fields, and the compound activity. The relationship between the compound activity and molecular field characteristics was established using partial least squares (PLS). In the first step, the training set compounds were cross verified using leave-one-out approach to obtain the cross-validated (q^2^) and optimal principal component (n) coefficients, and then the regression analysis was performed using no validation to obtain the standard error of estimate (SEE), non-cross-validated correlation coefficient (R^2^), and fischertest value (F). When the aforementioned parameters satisfied conditions, the training set was used to predict the test set. The correlation coefficient (r^2^_pred_) for test set predictions was calculated using the following formula to meet the external validation conditions, and then internal validation was performed. Q^2^, cSDEP, and dq^2^/dr^2^yy parameters were calculated to satisfy requirements to complete the construction of the double-activity 3D-QSAR model.

(8) $$r^{2}{}_{\mathrm{pred}}=1-\frac{\sum_{i=1}^{\mathrm{test}}{\left(y_{i}-\hat{y_{i}}\right)}^{2}}{\sum_{i=1}^{\mathrm{test}}{\left(y_{i}-\bar{y_{tr}}\right)}^{2}}$$

where $y_{i}$ is the experimental value of the test set, $\hat{y_{i}}$ is the estimated value of test set, and $\bar{y_{\mathrm{tr}}}$ is the mean experimental value of the training set.

### 2.4. Analysis of the Combined Toxicity Mechanism of QNs and Its Derivatives on Ryegrass

#### 2.4.1. Mixed Toxicity Index Method

The mixture toxic index method was first used by Könemann [49] to evaluate the combined toxic effect of multi-pollutants on fish. Ren et al. [35] used this method to evaluate the combined toxicity of residual QNs in the water environment. Therefore, the mixed toxicity index was adopted to evaluate the combined toxic effects of QNs on ryegrass in different soil types. The method is as follows:

(9) $${\mathrm{TU}}_{i}=\frac{C_{i}}{EC_{50}\left({\mathrm{IC}}_{50}\right)}$$

(10) $$M=\sum_{i=1}^{n}TU_{i}=\frac{C_{1}}{EC_{50}\left(IC_{50}\right)}+\frac{C_{2}}{EC_{50}\left(IC_{50}\right)}+\ldots +\frac{C_{n}}{EC_{n}\left(IC_{n}\right)}$$

(11) $$M_{0}=\frac{M}{{\left(TU_{i}\right)}_{\max}}$$

(12) $$M_{\mathrm{TI}}=1-\frac{\log M}{\log M_{0}}$$

where ${\mathrm{TU}}_{i}$ is the toxic unit of the component I in the mixture, $C_{i}$ is the concentration of the component i in the mixture, ${\mathrm{EC}}_{50}\left(IC_{50}\right)$ is the value of the ${\mathrm{EC}}_{50}$ or ${\mathrm{IC}}_{50}$, $M_{0}$ is the quotient between the sum of toxicity units of each component M and the maximum toxic unit of the mixture ${\left({\mathrm{TU}}_{i}\right)}_{\max}$, ${\left({\mathrm{TU}}_{i}\right)}_{\max}$ is the maximum value of ${\mathrm{TU}}_{i}$ in each mixture, and $M_{\mathrm{TI}}$ is the mixed toxicity index.

M_TI_ parameters were used as judgment criteria for combined toxic effects to characterize the types of the combined toxic effects of QNs on ryegrass, and judgment criteria given by Wang et al. [50] for various combined toxic effects were employed for the types of residual QNs on the combined toxic effects on ryegrass in three soil types (Table 1).

#### 2.4.2. Molecular Dynamics

Molecular dynamics was used to simulate the effect of different combinations of multiple QN mixtures on the degree of binding between QNs and two types of stress resistant enzymes (SOD and PPEP-2) in the farmland soil environment to verify and supplement the results of the combined toxicity of QNs on ryegrass based on the Gromacs software of the Dell PowerEdge R7425 server. The composite systems of SOD, PPEP-2 enzymes, ENR, and three ENR derivatives were placed in periodic 12 cubes with 15 nm side length. The GROMOS96-43a1 force field was used for molecule restriction, and Na^+^ was added to neutralize the system charge. The steepest gradient method was used for energy minimization simulation, the simulation time steps were set to 5,000,000 steps, the pressure bath was set to a constant standard atmospheric pressure of 1 bar, and the temperature was set to 300 k. Binding energy (G_bind_) was analyzed using the molecular mechanics Poisson–Bohzmann surface area (MMPBSA), and the effects of the combined toxicity of combinations of 11 QN mixtures on ryegrass were quantified. MMPBSA is a method to estimate the binding energy by post-processing of molecular dynamics trajectory. The solvent is treated as a continuous medium in the calculation, and many frames in the equilibrium trajectory are averaged based on the force field and implicit continuum model to consider the influence of temperature. The higher the absolute binding energy value, the stronger the interaction between enzymes and QN derivatives, indicating that the effect of the stress resistant enzymes can be completely exerted. The binding energy was calculated as follows:

(13) $$G_{\mathrm{bind}}=G_{\mathrm{complex}}-G_{\mathrm{free}-\mathrm{protein}}-G_{\mathrm{free}-\mathrm{ligand}}$$

(14) $$G=E_{\mathrm{gas}}-TS_{\mathrm{gas}}+G_{\mathrm{solvation}}$$

(15) $$G_{\mathrm{solvation}}=G_{\mathrm{polar}}+G_{\mathrm{nonpolar}}$$

(16) $$E_{\mathrm{gas}}=E_{\mathrm{bond}}+E_{\mathrm{angle}}+E_{\mathrm{dihedral}}+E_{\mathrm{vdw}}+E_{\mathrm{coulomb}}$$

where $G_{\mathrm{bind}}$ is the binding energy of the composite system, $G_{\mathrm{complex}}$ is the binding energy of the complex, $G_{\mathrm{free}-\mathrm{protein}}$ is the binding energy of the enzymes, $G_{\mathrm{free}-\mathrm{ligand}}$ is the binding energy of the molecules, G is the binding energy of the molecules in solution, T is the gas phase temperature, $E_{\mathrm{gas}}$ is the energy in the gas phase, $S_{\mathrm{gas}}$ is the entropy of the gas phase, $G_{\mathrm{solvation}}$ is the solvent binding energy, $G_{\mathrm{polar}}$ is the polar part contained in the solvent binding energy, $G_{\mathrm{nonpolar}}$ is the non-polar part contained in the solvent binding energy, $E_{\mathrm{bond}}$ is the bond interactions, $E_{\mathrm{angle}}$ is the bond angle interaction, $E_{\mathrm{dihedral}}$ is the interaction of dihedral angles, $E_{\mathrm{vdw}}$ is the Van der Waals action, $E_{\mathrm{coulomb}}$ is the coulomb electrostatic interaction.

## 3. Results and Analysis

### 3.1. Calculation of the Biotoxicity of QNs on Ryegrass Based on the Variation Weighting Method

DS software was used to molecularly dock 29 QN molecules with SOD and PPEP-2 enzymes and to calculate the LibDockScore (LDS). The variation weighting method was employed to integrate the two groups of molecular docking scores and obtain the CI values of QN molecules on ryegrass. Table 2 presents the specific calculation results.

### 3.2. Evaluation of the Double-Activity 3D-QSAR Model of the Biotoxicity of QNs on Ryegrass

The double-activity 3D-QSAR model indicated that the contribution rates of the steric and electrostatic fields of model were 75.6% and 24.4%, respectively, which showed that both the spatial and electronic distributions have an impact on the CI of QNs biotoxicity on ryegrass. In the double-activity 3D-QSAR model, the cross-validated (q^2^) and optimal principal component (n) coefficients are 0.767 (>0.5) and 3, respectively, which indicated that the model has good predictive ability [46]. The non-cross-validated correlation coefficient (R^2^), the standard error of estimate (SEE), and Fisher’s test value (F) of the model were 0.996 (>0.8), 0.008, and 614.264, respectively, indicating that the model has good fitting and predictive ability [51]. The (R^2^ − q^2^)/R^2^ of 22.992% < 25% indicated that no over fitting phenomenon occurred in the model [51]. The Q^2^, cSDEP, and dq^2^/dr^2^yy parameters of the scrambling stability test were 0.165, 0.119, and 1.679, respectively, which indicated that the model has good stability and predictive ability [52]. The external validation results obtained using formula (8) showed that the correlation coefficient (r^2^_pred_) was 0.967 (>0.6), which indicated that the model has good external predictive ability [53] (Table 3).

### 3.3. Molecular Modification of the Low Biotoxicity of QNs Derivatives on Ryegrass-3D Contour Maps

**Figure 2:**
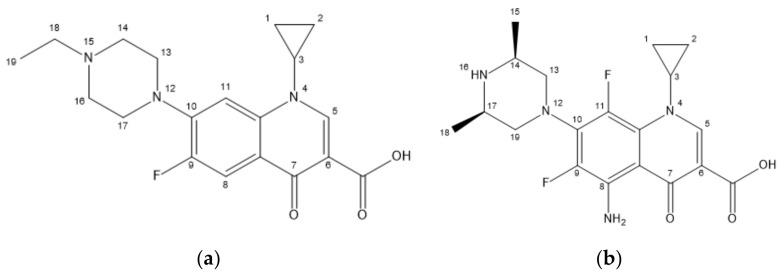
Target molecules (**a**) ENR (enrofloxacin) and (**b**) SPA (sparfloxacin).

**Figure 3:**
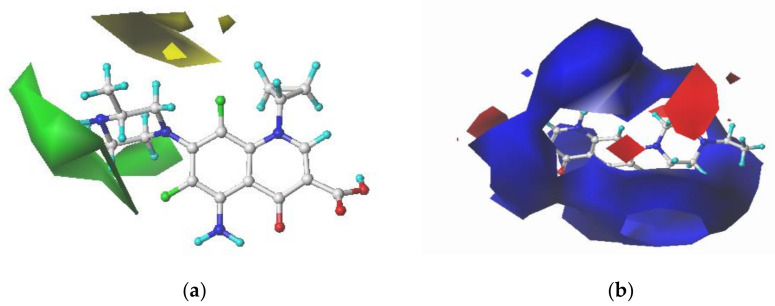
3D contour maps of the CoMFA model for enrofloxacin (ENR): (**a**) steric and (**b**) electrostatic fields.

**Figure 4:**
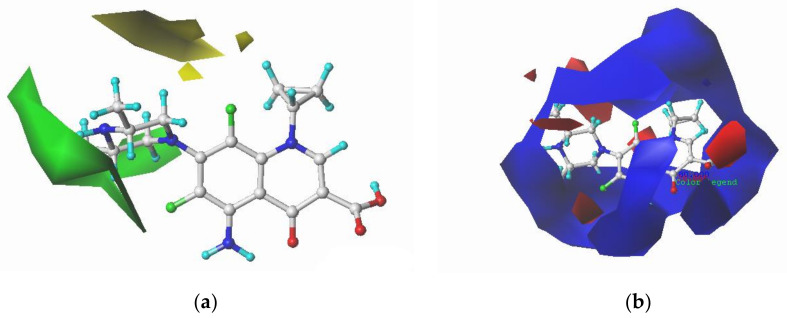
3D contour maps of the CoMFA model for sparfloxacin (SPA): (**a**) steric and (**b**) electrostatic fields.

ENR and SPA, third-generation fluoroquinolones with the largest CI, were selected as the target molecules for modification. Figure 2a,b illustrates the structural formulas of ENR and SPA. The force field distributions of the 3D contour maps of the CoMFA model are steric and electrostatic fields. In the steric field, the introduction of small and large groups near the green and yellow blocks, respectively, can reduce the biological toxicity. In the electrostatic field, the biotoxicity of compounds can be reduced by adding negative and positive groups near the blue and red blocks, respectively. Figure 3 and Figure 4 present the 3D contour maps of the CoMFA model of target molecules ENR and SPA. The types of the 3D contour map regions of the CoMFA model are standard deviation coefficient (stdev *-coeff), and the support and non-support contribution rates are 80% and 20%, respectively, in the default value [54].

The structure–activity relationship of QNs showed that the piperazine ring was the main structure of QNs to cause their antibacterial properties. The piperazine ring was believed to be the site directly acting on the DNA spiral enzyme of bacteria. The piperazine ring has a thick chain structure; thus, it can increase the antibacterial activity of QNs, block the efflux of components, and reduce the drug resistance [55].

By considering the information distribution characteristics presented in Figure 3 and Figure 4 and the structure–activity relationship of QNs, 9th and 19th sites of ENR and 8th and 17th sites of SPA were selected for modification. Small groups, namely –F, –Cl, and –Br, were introduced at 19th and 17th site of ENR and SPA, respectively. Weakly electronegative groups, namely –CH_3_, –CHO, –OH, –CH_2_OH, and –CH_2_CH_3_, were introduced at the 9th site of ENR; at the 8th site of SPA, negative groups, namely –NO, –NO_2_, –NF_2_, –CHO, and –COOH, were introduced. Through single and double substitution reactions, 23 ENR and 23 SPA derivatives were designed. The designed QNs derivatives were docked with SOD and Pro enzymes. Table 4 presents the molecular docking scores and their change rates.

The single effect characteristic ratio of the total scores between 17-chlorine-8-nitroso-SPA (D-14) and two enzymes (SOD and PPEP-2) was 4.17:5.08 (Table 4), which is consistent with the score weight (1:1) of the two enzymes observed in the biotoxicity double-activity model. Moreover, the predicted values of the double-activity model were consistent with those of the single-activity model, which indicated that the constructed double-activity 3D-QSAR model had certain accuracy and can be used to effectively select QNs modifications [56].

According to the analysis presented in Table 4, by considering ENR and its derivatives as examples, the change rates of the model predictive values of all the derivatives obtained by introducing –CHO, –CH_2_OH, and –CH_2_CH_3_ groups at 9th site of ENR are >10%, which is higher than those of ENR derivatives with –CH_3_ and –OH groups. In addition, for all the disubstituted combinations, the decrease in the predictive values of model for the derivatives with –OH substituted at 9th site is small. A similar phenomenon occurred in five derivatives with single substitution at 9th site, indicating that even though the electronegativity of the –OH group is strong, the biotoxicity of derivatives did not significantly improve, which may be related to the space structure of the –OH group. Furthermore, the electronegativity of the –CHO group is greater than that of the –CH_3_ group. Among the derivatives substituted with –CHO and –CH_3_ groups at the 9th site, the model predictive values of–CHO-substituted derivatives are lower than those of –CH_3_-substituted derivatives, except derivatives D-14 and D-15, in which the –Cl group was substituted at the 19th site.

### 3.4. Evaluation of the Environmental Friendliness and Functional Properties of ENR and SPA Derivatives

#### 3.4.1. Evaluation of the Environmental Friendliness

According to the information about the 3D contour maps of the double-activity 3D-QSAR model and QNs structure–activity relationship, the predicted CI values of 23 ENR and 23 SPA derivatives are lower than the CI values of the respective target molecules with the ranges of 3.09–20.11% and 2.06–15.25%, respectively (Table 4). This finding showed that the CI values of the derivatives designed using the variation weight method decreased compared with those of original target molecules, and the reduction degree of ENR derivatives is higher than that of SPA derivatives. Although the reduction of some derivatives is not sufficiently significant, it can be used as a theoretical reference for modifying environmentally friendly QN molecules.

The analysis results of the molecular docking scores obtained from the molecular docking of ENR and SPA derivatives with SOD and PPEP-2 enzymes (Table 4) showed that the total scores of all the ENR derivatives and SOD increased by 20.22–41.07%. However, the results of the molecular docking with PPEP-2 were not ideal, and only 7 derivatives, namely derivative-2 (D-2), derivative-3 (D-3), derivative-7 (D-7), derivative-10 (D-10), derivative-11 (D-11), derivative-12 (D-12), and derivative-17 (D-17), increased or remained constant. The docking results of SPA derivatives with SOD were satisfactory; however, derivative-13 (D-13) and derivative-18 (D-18) cannot be docked with SOD, and the score of derivative-15 (D-15) decreased. The increase in the scores of molecular docking between the remaining 20 SPA derivatives and SOD is 0.07–36.95%. Although the scores of some derivatives slightly increased, these scores can be used as a basis for further selection and evaluation. Approximately 1/2 of SPA derivatives can be successfully docked with PPEP-2, and the increase in total scores was 1.33–24.24%.

Some similarities and differences were found between the two enzymes. For example, docking scores for the derivative-2 (D-2) and derivative-3 (D-3), which were derived from ENR, and 12 derivatives, such as derivative-1 (D-1), derivative-2 (D-2), derivative-22 (D-22), which were derived from SPA, improved differently. Among them, the LDS of 17-chlorine-SPA (D-2) and two enzymes increased by >20%, indicating that the aforementioned two enzymes can bind more closely with these derivatives and induce their resistance. In addition, the docking of ENR and SPA derivatives and the two enzymes were different, and the docking result of derivatives and SOD was better than that of derivatives and PPEP-2, which may be related to the structure of the selected Pro enzyme. For the same protein type, the difference in the specific structure may lead to different results.

From the aforementioned analysis, 7 ENR derivatives, namely D-2, D-3, D-7, D-10, D-11, D-12, and D-17, and 11 SPA derivatives, including D-1, D-2, and D-3, were screened and subsequently analyzed.

Most QNs, which are not used by organisms, eventually enter the aquatic environment and lead to pollution [57]. However, they may simultaneously have a certain effect of algal removal. Studies have shown that QNs can cause acute toxicity to Scnedesmus obliquus [58]. Therefore, we evaluated the environmental friendliness of the aforementioned 7 ENR and 11 SPA derivatives. EPIWEB 4.1 software was used to predict bioaccumulation (log*K*_ow_), soil adsorbability (log*K*_oc_), and green algae toxicity, and the toxicity of green algae was given as the concentration required for 50% of maximal effect against green algae (EC_50_). We selected the phycocyanin (PDB ID: 4L1E) involved in cyanobacteria photosynthesis as the receptor from PDB database and ENR and SPA derivatives as ligands. Molecular docking was employed to calculate the molecular docking scores of ligand molecules and receptor proteins using the LibDock module in DS. These scores were used to characterize cyanobacterium toxicity. In addition, the biodegradability of derivatives was predicted using the model (Table 5).

Comparable to the target molecules, the 7 ENR and 11 SPA derivatives screened through the biotoxicity assessment showed the same variation trend for bioaccumulation and soil adsorbability, and no significant change was observed in biodegradability (Table 5). The bioaccumulation and soil adsorbability of the 7 ENR derivatives decreased in the range of −238.57% to −21.43% and −644.93% to −40.10%, respectively. Among these derivatives, these two properties of D-7, D-10, D-11, D-12, and D-17 were substantially improved. The biodegradability of all the 7 ENR derivatives decreased; however, with a minimum decrease of 3.10%, the maximum decrease was only 6.83%, which suggested that the change was insignificant. Among the 11 SPA derivatives, only five molecules (D-1, D-2, D-3, D-8 and D-23) simultaneously met bioaccumulation and soil adsorbability requirements, which decreased by −39.29% to −10.71% and −30.55% to −8.34%, respectively. The variation range of biodegradability data of these five derivatives indicated that the biodegradability of D-1 increased by 0.78% and that of other four derivatives remained unchanged.

In addition, from the data of QNs toxicity to algae, we found that ENR and SPA derivatives exhibited a considerably better effect of toxicity promotion to green algae than that to cyanobacteria. This finding showed that the green algae removal capacity of derivatives was enhanced after modification, which is similar to the results of Ebert et al. [59] who found that enrofloxacin was highly toxic towards green algae, while ciprofloxacin was more toxic to cyanobacteria. The toxicity of 9-methanol-ENR (D-7), 19-fluorine-9-methanol-ENR (D-12), and 19-chlorine-9-methanol-ENR (D-17) towards green algae and cyanobacteria increased to 513.93% and 4.15%, respectively, 1391.81% and 0.78%, respectively, and 845.10% and 6.48%, respectively. The increased ratio range of the toxicity of SPA derivatives (D-1, D-2, D-3, D-8, and D-23) to green algae was 47.49–153.68%, among which the increase rates of D-1 and D-23 were >100%. This finding indicated that the derivatives had a strong capacity to remove green algae after modification. The toxicity of 17-bromine-SPA (D-3) and 17-bromine-8-carboxyl-SPA (D-23) towards cyanobacteria increased by 3.26% and 3.06%, respectively, and the toxicity of the other three SPA derivatives towards cyanobacteria remained unchanged. Although the data showed that the enhancement of cyanobacteria toxicity of the derivatives was not sufficiently ideal, it could still be used to remove green algae and cyanobacteria, which can be a novel method for bloom control.

As the basis for functional evaluation, 3 ENR derivatives (D-7, D-12, and D-17) and 5 SPA derivatives (D-1, D-2, D-3, D-8, and D-23) with environmental friendliness were screened.

#### 3.4.2. Evaluation of the Functional Properties

QNs are a class of antibacterial drugs with considerable potential which can be employed in clinical care and veterinary medicines [60]. Salmonella typhimurium is a type of pathogenic bacteria, which belongs to typical Gram-negative bacteria, that can cause food poisoning [61]. The HQSAR model of genotoxicity of QNs towards salmonella typhurium established by Zhao et al. [62] was used to predict the bacterial genotoxicity, expressed as pLOEC, of the aforementioned derivatives (Table 6).

Table 6 shows that the genotoxicity of SPA derivatives is slightly higher than that of ENR derivatives. The genotoxicity of all five SPA derivatives increased by 2.23–7.84% and the maximum genotoxicity was observed for 17-fluorine-SPA. The genotoxicity of 9-methanol-ENR (D-7) moderately increased, and that of 19-fluorine-9-hydroxy-ENR (D-11), 19-fluorine-9-methanol-ENR (D-12), and 19-chlorine-9-methanol-ENR (D-17) decreased slightly, that is, the function did not change substantially. Therefore, 3 ENR and 5 SPA derivatives with higher genotoxicity, lower biotoxicity, and environmental friendliness were screened out, which provided considerable theoretical guidance for the design of environmentally friendly antimicrobial drugs.

### 3.5. Analysis of the Mechanism of the Combined Toxicity of QNs on Ryegrass Based on the Mixture Toxic Index Method and Molecular Dynamics

#### 3.5.1. Analysis Based on the Mixed Toxicity Index Method

The diversity of antibiotic applications leads to different degrees of combined pollution and many studies have focused on the toxicity of combined pollution of antibiotics at home and abroad. Mao et al. [63] studied the combined toxicity actions of animal sulphonamideson crops and found that the combined pollution of sulphadiazine (SD) and sulphamonomethoxine showed synergistic and antagonistic actions at low and high concentrations, respectively. Shi et al. [64] investigated the negative effects of binary QN mixtures, such as levofloxacin and enrofloxacin, on the relative luminescence intensity of vibrio qinghaiensissp-Q67. Yang et al. [65] reported the addition and synergistic effect of the binary mixtures of 12 antibiotics, including ofloxacin, on freshwater algae. Ryegrass is a type of forage grass with high growth capacity and adaptability which can be cultivated artificially or grown naturally in different environmental regions such as farmland, garden, woodland, or urban lawn [66]. We calculated the combined toxic effects of QN mixtures before and after modification in different soil environments and on naturally growing ryegrass in soil to screen QN derivatives with potentially low environmental risks. Because the derivatives remain available at the theoretical level and are not being produced and used, the real residual concentrations in the soil were not obtained. We selected the residual concentrations of ENR present in three different soil types from literature [67] as that of ENR and its derivatives, 9-methanol-ENR, 19-fluorine-9-methanol-ENR, and 19-chlorine-9-methanol-ENR. The biotoxicity of QNs on ryegrass in soil was characterized through the predicted values of the double-activity 3D-QSAR model of ENR and the aforementioned three ENR derivatives (Table 7). Moreover, the mixed toxicity index, M_TI_, was calculated by following the mixture toxic index method, and was used to determine the combined toxicity of QNs on ryegrass before and after modification in farmland, garden, and woodland soil types (Table 8 and Table 9).

According to the judgment criteria (Table 1), we discussed the combined toxicity of ENR (A) and its three derivatives, namely 9-methanol-ENR (B), 19-fluorine-9-methanol-ENR (C), and 19-chlorine-9-methanol-ENR (D), in three soil types, namely farmland, garden, and woodland. Because this study explored the toxicity changes in QN molecules before and after modification, the horizontal comparison principle was adopted to compare the changes in combined toxicity of different derivatives and ENR based on the original ENR molecule, and the combined toxicity of multiple mixtures was considered.

In the farmland environment, the M_TI_ values of QNs were between 0 and 1, indicating that the combined toxicity of ENR and its derivatives on ryegrass was partial additive action. First, based on the target molecule ENR (A), in binary mixtures, the M_TI_ value of the A * B combination was lower than that of A * C and A * D combinations, with a difference of 19.98% and 15.10%, respectively, which indicated that the contribution of derivative B to reduce the combined toxicity on ryegrass was higher than that of C and D. In addition, based on the A * B combination, the M_TI_ values of ternary mixtures A * B * C and A * B * D were similar and decreased by 73.51% compared with that of binary mixture A * B, which indicated that both C and D derivatives had similar contributions to combined toxicity and can reduce it. Based on A * C, the M_TI_ values of ternary mixtures A * C * D and A * B * C decreased by 80.47% and 108.19%, respectively, suggesting that both derivatives B and D can reduce the combined toxicity of mixtures, and the degree of reduction of B was high. Based on A * D, the M_TI_ values of ternary mixtures A * C * D and A * B * D decreased by 73.12% and 100.10%, respectively, indicating that both B and C derivatives played a role in the reduction of the combined toxicity of mixtures, and the effect of B was more significant than that of C. According to C * D, the combined toxicity of B * C * D decreased by 83.33%, which suggested that B can evidently reduce the combined toxicity of mixtures. Moreover, the combined pollution toxicity of sole derivatives was lower than that of combinations having A for the same amounts of combinations. The combined toxicity of quaternion mixture A * B * C * D was the lowest among the 11 groups, and its M_TI_ value was 0.079. Furthermore, the toxic index of mixtures of 11 combinations simulated in the farmland soil environment was lower than the CI values of ENR (1.069) on ryegrass, which indicated that the combined toxicity of QNs on ryegrass after modification in the farmland soil environment decreased. This analysis showed that when QNs were present in the farmland, the type of combined toxicity was partial additive action, and the combined toxicity of the quaternary mixture was lower than that of the binary and ternary mixtures, that is, the modified QN derivatives can effectively reduce the combined toxicity of ryegrass.

In the garden and woodland soil, the M_TI_ values of QNs were >1, indicating that the combined toxicity of ENR and its derivatives was synergistic, and the M_TI_ values of all the 22 simulated combinations were higher than the biotoxicity values of ENR. Based on A * B, the combined toxicity of ternary mixtures A * B * C and A * B * D decreased by approximately 73%. Based on A * C, the combined toxicity of ternary mixtures A * C * D and A * B * C decreased by approximately 72%. Based on A * D, the combined toxicity of ternary mixtures A * C * D and A * B * D decreased by about 73%. These findings indicated that derivatives B, C, and D can reduce the combined toxicity of mixtures and can contribute to a similar degree of reduction. The M_TI_ values of A * B * C * D in two soil environments were 1.550 and 2.008, and the combined toxicity of A * B * C * D was lower than that of the ternary mixtures, whereas that of ternary mixtures was lower than that of binary mixtures. Furthermore, the combined toxicity of sole derivatives was lower than that of combinations having ENR, which showed that the modified derivatives were more environmentally friendly than ENR combinations. This finding is consistent with the aforementioned environmental risk evaluation results. Under various types of synergistic effects, we compared the combined toxicity of different combinations in the same soil environment and found that the combined toxicity tended to decrease smoothly with an increase in the pollutant quantity, which may be related to the interference of external natural factors, such as the type of parent materials of soils, pH, and organic matter. Zhang et al. [68] analyzed the contents of antibiotics in the Zhangjiakou Grape Park soil and found that pH was significantly negatively correlated with the adsorption capacity of QNs in the soil, and organic matter might weaken this correlation.

In conclusion, the application of modified QN derivatives to different soil types can lead to a decrease in the risk of environmental ecology. The risk of environmental ecology is the lowest in the farmland soil environment, and the biotoxicity and combined toxicity of naturally growing or artificial planted perennial ryegrass significantly decreased, which is consistent with the derivative evaluation results.

#### 3.5.2. Analysis Based on Molecular Dynamics

By considering the farmland soil environment as an example, the combined toxicity of the 11 combinations of ENR and its three derivatives on ryegrass was calculated through molecular dynamics, and the binding energy of the composite system (Table 10) was characterized as an indicator of the combined toxicity of ryegrass.

The absolute binding energies of ternary mixtures B * C * D and A * C * D are 59.85% and 55.23%, respectively, higher than those of binary mixture C * D, which indicated that derivative B has a higher capacity of binding to enzymes than A, i.e., derivative B can reduce the combined toxicity of mixtures (Table 10). The absolute binding energy of A * B * D was 24.68% higher than that of A * B, indicating that the addition of derivative D could lead the mixture to bind considerably closely with the enzymes, that is, derivative D can highly reduce the combined toxicity. The absolute binding energy of A * B * C and A * C * D was higher than that of A * C, with a difference of 65.50% and 48.41%, respectively, indicating that both B and D can bind with the enzymes considerably closely, and the binding capacity of B was higher than that of D, i.e., derivative B can evidently reduce the combined toxicity. The absolute binding energy of A * B * D and A * C * D was 54.72% and 6.54%, respectively, higher than that of A * D, respectively, which indicated that both B and C can closely bind with the enzymes, and the binding capacity of B was higher than that of C, i.e., derivative B can reduce the combined toxicity. In addition, among the 11 combinations (Table 10), the absolute binding energy of A * B * C * D was the highest, which corresponded to the minimum M_TI_ value, indicating that the combined toxicity of A * B * C * D was the lowest. Thus, the results of the molecular dynamics simulation of the combined toxicity of QNs on ryegrass are consistent with the evaluation results obtained from the mixed toxicity index method, thereby further verifying and supplementing the result that the application of modified QNs derivatives to the farmland soil environment can substantially reduce the combined toxicity of perennial ryegrass and indicating that the designed QN derivatives can reduce the ecological risk in the soil environment.

## 4. Conclusions

By using the variation weighting method combined with molecular docking, the double-activity 3D-QSAR model of the biotoxicity of QN molecules on ryegrass was constructed and successfully employed for the modification of QN derivatives with low biotoxicity, high genotoxicity, and environmental friendliness. This model was used for the mechanism verification of the combined toxicity of QNs on ryegrass in farmland, garden, and woodland environments. The model provides a theoretical reserve for the replacement of QN antibiotics to achieve environmental and plant friendliness.

## Figures and Tables

**Table 1:** Criteria for the types of combined toxic action in the environment.

Synergistic Effect	Simple Additive Action	Independent Action	Antagonism	Partial Additive Action
M_TI_ > 1	M_TI_ = 1	M_TI_ = 0	M_TI_ < 0	0 < M_TI_ < 1

**Table 2:** Calculation of CI of the biotoxicity of QNs (quinolones) on ryegrass.

No.	Compounds	Abbreviations	LibDockScore		Comprehensive Value (CI)
			1B06	6FPC	
1	Difloxacin	DIF	73.21	69.19	1.074
2	Enrofloxacin	ENR	68.72	72.86	1.069
3	Norfloxacin	NOR	64.63	62.14	0.956
4	Lomefloxacin	LOM	64.07	59.99	0.936
5	Levofloxacin	LEV	65.51	71.06	1.032
6	Pefloxacin	PEF	69.37	66.10	1.022
7	Fleroxacin	FLE	63.24	65.18	0.969
8	Ciprofloxacin	CIP	68.95	64.63	1.007
9	Balofloxacin	BAL	49.90	48.84	0.745
10	Marbofloxacin	MAR	66.70	69.40	1.028
11	Pipemidic acid	PIP	69.90	66.52	1.029
12	Cinoxacin	CIN	69.14	69.79	1.049
13	Enoxacin	ENO	67.21	65.650	1.002
14	Danofloxacin	DAN	74.21	74.57	1.123
15	Gatifloxacin	GAT	52.29	51.07	0.780
16	Ofloxacin	OFL	65.51	71.06	1.032
17	Rufloxacin	RUF	59.46	64.04	0.933
18	Pazufloxacin	PAZ	73.50	69.93	1.082
19	Nadifloxacin	NAD	65.95	69.04	1.019
20	Moxifloxacin	MOX	56.09	57.62	0.858
21	Sparfloxacin	SPA	72.78	68.90	1.069
22	Sarafloxacin	SAR	65.93	62.12	0.966
23	Amifloxacin	AMI	68.55	67.45	1.026
24	Besifloxacin	BES	64.10	56.74	0.911
25	Clinafloxacin	CLI	64.58	69.36	1.012
26	Grepafloxacin	GRE	68.90	66.78	1.024
27	Orbifloxacin	ORB	66.90	67.14	1.012
28	Sitafloxacin	SIT	69.50	61.53	0.987
29	Temafloxacin	TEM	72.64	68.20	1.062

**Table 3:** Double-activity 3D-QSAR model parameters of QNs biotoxicity.

Model	q^2^	n	R^2^	SEE	F	Q^2^	cSDEP	dq^2^/dr^2^yy	r^2^_pred_
CoMFA	0.767	3	0.996	0.008	614.264	0.165	0.119	1.679	0.967

**Table 4:** Evaluation of biotoxicity of ENR and SPA derivatives.

No.	Compounds	CoMFA Model		LibDockScore			
		Pred.	Relative Error (%)	1B06	Relative Error (%)	6FPC	Relative Error (%)
2	ENR	1.069		68.72		72.86	
Derivative-1	19-Fluorine-ENR	0.901	−15.72	85.00	23.69	51.06	−22.93
Derivative-2	19-Chlorine-ENR	1.035	−3.18	82.61	20.22	77.36	6.17
Derivative-3	19-Bromine-ENR	1.036	−3.09	88.54	28.84	78.05	7.12
Derivative-4	9-Methyl-ENR	1.005	−5.99	91.14	32.63	56.67	−22.23
Derivative-5	9-Aldehyde-ENR	0.861	−19.46	91.92	33.77	67.11	−7.90
Derivative-6	9-Hydroxy-ENR	1.018	−4.77	89.32	29.98	64.93	−10.89
Derivative-7	9-Methanol-ENR	0.858	−19.74	92.96	35.28	72.65	−0.30
Derivative-8	9-Ethyl-ENR	0.903	−15.53	85.76	24.79	64.04	−12.11
Derivative-9	19-Fluorine-9-Methyl-ENR	1.024	−4.21	88.62	28.96	60.54	−16.91
Derivative-10	19-Fluorine-9-Aldehyde-ENR	0.876	−18.05	92.03	33.92	70.03	−3.89
Derivative-11	19-Fluorine-9-Hydroxy-ENR	1.024	−4.21	92.69	34.88	72.17	−0.95
Derivative-12	19-Fluorine-9-Methanol-ENR	0.878	−17.87	93.43	35.96	68.80	−5.57
Derivative-13	19-Fluorine-9-Ethyl-ENR	0.923	−13.66	95.77	39.36	61.19	−16.02
Derivative-14	19-Chlorine-9-Methyl-ENR	0.854	−20.11	88.21	28.37	60.22	−17.35
Derivative-15	19-Chlorine-9-Aldehyde-ENR	0.955	−10.66	92.81	35.06	63.79	−12.46
Derivative-16	19-Chlorine-9-Hydroxy-ENR	1.027	−3.93	86.93	26.50	63.16	−13.31
Derivative-17	19-Chlorine-9-Methanol-ENR	0.873	−18.33	96.94	41.07	70.00	−3.93
Derivative-18	19-Chlorine-9-Ethyl-ENR	0.899	−15.90	93.06	35.42	55.60	−23.70
Derivative-19	19-Bromine-9-Methyl-ENR	1.028	−3.84	92.17	34.13	64.85	−11.00
Derivative-20	19-Bromine-9-Aldehyde-ENR	0.876	−18.05	91.44	33.05	64.85	−11.00
Derivative-21	19-Bromine-9-Hydroxy-ENR	1.027	−3.93	88.81	29.24	65.76	−9.76
Derivative-22	19-Bromine-9-Methanol-ENR	0.872	−18.43	92.26	34.26	66.85	−8.26
Derivative-23	19-Bromine-9-Ethyl-ENR	0.894	−16.37	87.82	27.80	64.09	−12.04
21	SPA	1.069		72.78		68.90	
Derivative-1	17-Fluorine-SPA	1.000	−6.45	96.18	32.15	75.74	9.93
Derivative-2	17-Chlorine-SPA	1.038	−2.90	99.68	36.95	85.22	23.69
Derivative-3	17-Bromine-SPA	1.036	−3.09	91.50	25.73	82.42	19.63
Derivative-4	8-Nitroso-SPA	0.970	−9.26	76.31	4.84	74.43	8.03
Derivative-5	8-Nitro-SPA	1.014	−5.14	83.25	14.38	72.34	5.00
Derivative-6	8-NF_2_-SPA	1.025	−4.12	74.68	2.61	57.64	−16.34
Derivative-7	8-Aldehyde-SPA	1.032	−3.46	98.72	35.64	78.03	13.26
Derivative-8	8-Carboxyl-SPA	0.906	−15.25	91.22	25.33	76.17	10.56
Derivative-9	17-Fluorine-8-Nitroso-SPA	1.013	−5.24	99.80	37.12	68.22	−0.98
Derivative-10	17-Fluorine-8-Nitro-SPA	0.966	−9.64	83.04	14.09	61.17	−11.21
Derivative-11	17-Fluorine-8-NF_2_-SPA	0.965	−9.73	75.12	3.21	62.79	−8.86
Derivative-12	17-Fluorine-8-Aldehyde-SPA	0.958	−10.38	95.98	31.87	/	/
Derivative-13	17-Fluorine-8-Carboxyl-SPA	1.024	−4.21	/	/	76.72	11.36
Derivative-14	17-Chlorine-8-Nitroso-SPA	1.018	−4.77	87.27	19.91	85.60	24.24
Derivative-15	17-Chlorine-8-Nitro-SPA	1.043	−2.43	69.73	−4.19	55.77	−19.05
Derivative-16	17-Chlorine-8-NF_2_-SPA	1.024	−4.21	73.36	0.80	58.68	−14.82
Derivative-17	17-Chlorine-8-Aldehyde-SPA	1.009	−5.61	77.98	7.14	70.75	2.69
Derivative-18	17-Chlorine-8-Carboxyl-SPA	0.917	−14.22	/	/	/	/
Derivative-19	17-Bromine-8-Nitroso-SPA	1.031	−3.55	96.83	33.04	59.26	−13.99
Derivative-20	17-Bromine-8-Nitro-SPA	1.043	−2.43	72.83	0.07	63.20	−8.26
Derivative-21	17-Bromine-8-NF_2_-SPA	1.013	−5.24	73.18	0.55	55.72	−19.13
Derivative-22	17-Bromine-8-Aldehyde-SPA	1.047	−2.06	95.45	31.14	69.82	1.33
Derivative-23	17-Bromine-8-Carboxyl-SPA	1.009	−5.61	98.26	35.00	82.88	20.30

**Table 5:** Evaluation of environmental friendliness for ENR and SPA derivatives.

No.	Compounds	Bioaccumulation		Soil Adsorbability		Biodegradability		Green Algae Toxicity		Cyanobacteria Toxicity	
		log*K*_ow_	Relative Error (%)	log*K*_oc_	Relative Error (%)	Pred.	Relative Error (%)	EC_50_	Relative Error (%)	LDS	Relative Error (%)
2	ENR	0.700		0.207		3.194		989.25		102.61	
Derivative-2	19-Chlorine-ENR	0.460	−34.29	0.074	−64.25	3.090	−3.26	1525.53	54.21	93.48	−8.90
Derivative-3	19-Bromine-ENR	0.550	−21.43	0.124	−40.10	3.095	−3.10	1477.53	49.36	83.06	−19.05
Derivative-7	9-Methanol-ENR	−0.420	−160.00	−0.824	−498.07	3.066	−4.01	6073.24	513.93	106.87	4.15
Derivative-10	19-Fluorine-9-Aldehyde-ENR	−0.330	−147.14	−0.592	−385.99	3.008	−5.82	5337.51	439.55	87.82	−14.42
Derivative-11	19-Fluorine-9-Hydroxy-ENR	−0.530	−175.71	−0.306	−247.83	3.031	−5.10	7075.86	615.28	87.96	−14.28
Derivative-12	19-Fluorine-9-Methanol-ENR	−0.970	−238.57	−1.128	−644.93	2.976	−6.83	14,757.63	1391.81	103.42	0.78
Derivative-17	19-Chlorine-9-Methanol-ENR	−0.650	−192.86	−0.951	−559.42	3.033	−5.04	9349.34	845.10	109.27	6.48
21	SPA	1.400		0.995		3.089		356.04		81.62	
Derivative-1	17-Fluorine-SPA	0.850	−39.29	0.691	−30.55	3.113	0.78	864.68	142.86	80.84	−0.94
Derivative-2	17-Chlorine-SPA	1.160	−17.14	0.863	−13.27	3.084	−0.16	546.64	53.53	76.85	−5.84
Derivative-3	17-Bromine-SPA	1.250	−10.71	0.912	−8.34	3.052	−1.20	525.12	47.49	84.28	3.26
Derivative-4	8-Nitroso-SPA	2.180	55.71	1.427	43.42	3.240	4.89	105.17	−70.46	66.40	−18.65
Derivative-5	8-Nitro-SPA	2.130	52.14	1.640	64.82	2.951	−4.47	118.85	−66.62	65.15	−20.17
Derivative-7	8-Aldehyde-SPA	2.030	45.00	1.136	14.17	3.081	−0.26	133.87	−62.40	77.32	−5.27
Derivative-8	8-Carboxyl-SPA	1.090	−22.14	0.845	−15.08	3.031	−1.88	618.44	73.70	77.64	−4.87
Derivative-14	17-Chlorine-8-Nitroso-SPA	1.950	39.29	1.300	30.65	3.078	−0.36	161.20	−54.72	68.43	−16.15
Derivative-17	17-Chlorine-8-Aldehyde-SPA	1.790	27.86	1.003	0.80	3.047	−1.36	205.21	−42.36	77.49	−5.05
Derivative-22	17-Bromine-8-Aldehyde-SPA	1.880	34.29	1.053	5.83	3.045	−1.42	196.55	−44.80	83.50	2.31
Derivative-23	17-Bromine-8-Carboxyl-SPA	0.950	−32.14	0.768	−22.81	3.046	−1.39	903.20	153.68	84.11	3.06

**Table 6:** Predicted pLOEC values of ENR and SPA derivatives.

No.	Compounds	Genotoxicity	
		pLOEC	Relative Error (%)
2	ENR	8.839	
Derivative-7	9-Methanol-ENR	8.854	0.17
Derivative-12	19-Fluorine-9-Methanol-ENR	8.397	−5.00
Derivative-17	19-Chlorine-9-Methanol-ENR	8.405	−4.91
21	SPA	7.486	
Derivative-1	17-Fluorine-SPA	8.073	7.84
Derivative-2	17-Chlorine-SPA	7.939	6.05
Derivative-3	17-Bromine-SPA	8.035	7.33
Derivative-8	8-Carboxyl-SPA	7.653	2.23
Derivative-23	17-Bromine-8-Carboxyl-SPA	7.870	5.13

**Table 7:** QNs concentration and toxicity data in three soil types.

Types	QNs and Derivatives	Levels (μg/kg)	Biotoxicity Values
Farmland	ENR	0.773 [68]	1.069
	9-Methanol-ENR		0.858
	19-Fluorine-9-Methanol-ENR		0.878
	19-Chlorine-9-Methanol-ENR		0.873
Garden	ENR	0.110 [68]	1.069
	9-Methanol-ENR		0.858
	19-Fluorine-9-Methanol-ENR		0.878
	19-Chlorine-9-Methanol-ENR		0.873
Woodland	ENR	0.060 [68]	1.069
	9-Methanol-ENR		0.858
	19-Fluorine-9-Methanol-ENR		0.878
	19-Chlorine-9-Methanol-ENR		0.873

**Table 8:** Combined toxicity of QNs on ryegrass before and after modification in three soil types.

Types	Groups	Combination ^a^	Parameters							
			TU					M	M_0_	M_TI_
			A	B	C	D	max			1-logM/logM_0_
Farmland	1	A * B	0.723	0.901			0.901	1.624	1.803	0.177
	2	A * C	0.723		0.880		0.880	1.604	1.821	0.212
	3	A * D	0.723			0.885	0.885	1.609	1.817	0.204
	4	B * C		0.901	0.880		0.901	1.781	1.977	0.153
	5	B * D		0.901		0.885	0.901	1.786	1.983	0.152
	6	C * D			0.880	0.885	0.885	1.766	1.994	0.176
	7	A * B * C	0.723	0.901	0.880		0.901	2.504	2.780	0.102
	8	A * B * D	0.723	0.901		0.885	0.901	2.509	2.785	0.102
	9	A * C * D	0.723		0.880	0.885	0.885	2.489	2.811	0.118
	10	B * C * D		0.901	0.880	0.885	0.901	2.667	2.960	0.096
	11	A * B * C * D	0.723	0.901	0.880	0.885	0.901	3.390	3.763	0.079
Garden	1	A * B	0.103	0.128			0.128	0.231	1.803	3.486
	2	A * C	0.103		0.125		0.125	0.228	1.821	3.464
	3	A * D	0.103			0.126	0.126	0.229	1.817	3.470
	4	B * C		0.128	0.125		0.128	0.253	1.977	3.013
	5	B * D		0.128		0.126	0.128	0.254	1.983	3.001
	6	C * D			0.125	0.126	0.126	0.251	1.994	3.001
	7	A * B * C	0.103	0.128	0.125		0.128	0.356	2.780	2.009
	8	A * B * D	0.103	0.128		0.126	0.128	0.357	2.785	2.005
	9	A * C * D	0.103		0.125	0.126	0.126	0.354	2.811	2.004
	10	B * C * D		0.128	0.125	0.126	0.128	0.379	2.960	1.893
	11	A * B * C * D	0.103	0.128	0.125	0.126	0.128	0.482	3.763	1.550
Woodland	1	A * B	0.056	0.070			0.070	0.126	1.803	4.515
	2	A * C	0.056		0.068		0.068	0.124	1.821	4.475
	3	A * D	0.056			0.069	0.069	0.125	1.817	4.485
	4	B * C		0.070	0.068		0.070	0.138	1.977	3.902
	5	B * D		0.070		0.069	0.070	0.139	1.983	3.886
	6	C * D			0.068	0.069	0.069	0.137	1.994	3.879
	7	A * B * C	0.056	0.070	0.068		0.070	0.194	2.780	2.602
	8	A * B * D	0.056	0.070		0.069	0.070	0.195	2.785	2.597
	9	A * C * D	0.056		0.068	0.069	0.069	0.193	2.811	2.591
	10	B * C * D		0.070	0.068	0.069	0.070	0.207	2.960	2.451
	11	A * B * C * D	0.056	0.070	0.068	0.069	0.070	0.263	3.763	2.008

**^a^** A: ENR; B: 9-methanol-ENR; C: 19-fluorine-9-methanol-ENR; D: 19-chlorine-9-methanol-ENR; *: compounds combination symbol.

**Table 9:** Judgment of the combined toxicity of QNs in three soil types.

Types	Combinations	Evaluation System	Evaluation Results	Type of Combined Action
Farmland	11	M_TI_	0.079~0.212	Partial additive action
Garden	11		1.550~3.486	Synergistic
Woodland	11		2.008~4.515	Synergistic

**Table 10:** Binding energy between QNs and ryegrass toxicity receptors (SOD and Pro) before and after modification.

Situation	Groups	Combination	Binding Energy (kcal/mol)	M_TI_
Farmland	1	A * B	−239.51	0.177
	2	A * C	−79.48	0.212
	3	A * D	−143.99	0.204
	4	B * C	−187.77	0.153
	5	B * D	−214.18	0.152
	6	C * D	−68.97	0.176
	7	A * B * C	−230.38	0.102
	8	A * B * D	−317.98	0.102
	9	A * C * D	−154.06	0.118
	10	B * C * D	−171.80	0.096
	11	A * B * C * D	−331.98	0.079

*: compounds combination symbol.

## References

[1] Rodriguez-Mozaz S., Vaz-Moreira I., Varela D.G.S., Llorca M., Barceló D., Schubert S., Berendonk T.U., Michael-Kordatou I., Fatta-Kassinos D., Martinez J.L. (2020). Antibiotic residues in final effluents of European wastewater treatment plants and their impact on the aquatic environment. Environ. Int..

[2] Kümmerer K. (2009). Antibiotics in the aquatic environment—A review-Part I. Chemosphere.

[3] Aldred K.J., Kerns R.J., Osheroff N. (2014). Mechanism of Quinolone Action and Resistance. Biochemistry.

[4] Ghaly H., Jörns A., Rustenbeck I. (2014). Effect of fluoroquinolones on mitochondrial function in pancreatic beta cells. Eur. J. Pharm. Sci..

[5] Zhang G.D., Dong W.P., Liu X.H., Liu Y., Zhang L.L., Yan X.S., Wang W.L. (2018). Occurrence, fate and risk assessment of antibiotics in water environment of China. Environ. Chem..

[6] Picó Y., Andreu V. (2006). Fluoroquinolones in soil-risks and challenges. Anal. Bioanal. Chem..

[7] Ji X.L., Shen Q.H., Liu F., Ma J., Xu G., Wang Y.L., Wu M.H. (2012). Antibiotic resistance gene abundances associated with antibiotics and heavy metals in animal manures and agricultural soils adjacent to feedlots in Shanghai; China. J. Hazard. Mater..

[8] Gao L.H., Shi Y.L., Li W.H., Niu H.Y., Liu J.M., Cai Y.Q. (2012). Occurrence of antibiotics in eight sewage treatment plants in Beijing, China. Chemosphere.

[9] Yan C.X., Yang Y., Zhou J.L., Liu M., Nie M.H., Shi H., Gu L.J. (2013). Antibiotics in the surface water of the Yangtze Estuary: Occurrence, distribution and risk assessment. Environ. Pollut..

[10] Vasudevan D., Bruland G.L., Torrance B.S., Upchurch V.G., MacKay A.A. (2009). pH-dependent ciprofloxacin sorption to soils: Interaction mechanisms and soil factors influencing sorption. Geoderma.

[11] Yang L., Wu L.H., Liu W.X., Huang Y.J., Luo Y.M., Christie P. (2018). Dissipation of antibiotics in three different agricultural soils after repeated application of biosolids. Environ. Sci. Pollut. Res..

[12] Morales-Muñoz S., Luque-García J.L., LuquedeCastro M.D. (2004). Continuous microwave-assisted extraction coupled with derivatization and fluorimetric monitoring for the determination of fluoroquinolone antibacterial agents from soil samples. J. Chromatogr. A.

[13] Golet E.M., Strehler A., Alder A.C., Giger W. (2002). Determination of Fluoroquinolone Antibacterial Agents in Sewage Sludge and Sludge-Treated Soil Using Accelerated Solvent Extraction Followed by Solid-Phase Extraction. Anal. Chem. (Wash.).

[14] Wang Y.M. (2019). Toxic Effects of Cu and Fluoroquinolone Antibiotics on Ryegrass. Master’s Thesis.

[15] Lu J.Y., Li X., Yang Y.T., Nie X.P. (2007). Toxic effects of nutylated hydroxyanisole and norfloxacin on aquatic organisms. Ecol. Sci..

[16] Pfeifer M., Martis M., Asp T., Mayer K.F.X., Lübberstedt T., Byrne S., Frei U., Studer B. (2013). The Perennial Ryegrass Genome Zipper: Targeted Use of Genome Resources for Comparative Grass Genomics. Plants Physiol. (Bethesda).

[17] Günther T., Dornberger U., Fritsche W. (1996). Effects of ryegrass on biodegradation of hydrocarbons in soil. Chemosphere.

[18] Peach M.E., Hicks Pries C.E., Friedland A.J. (2021). Plants and earthworms control soil carbon and water quality trade-offs in turfgrass mesocosms. Sci. Total Environ..

[19] Cyriac D., Hofmann R.W., Stewart A., Sathish P., Winefield C.S., Moot D.J. (2018). Intraspecific differences in long-term drought tolerance in perennial ryegrass. PLoS ONE.

[20] Azanu D., Styrishave B., Darko G., Weisser J.J., Abaidoo R.C. (2018). Occurrence and risk assessment of antibiotics in water and lettuce in Ghana. Sci. Total Environ..

[21] Hu X.Q., Zhou Q.X., Luo Y. (2010). Occurrence and source analysis of typical veterinary antibiotics in manure, soil, vegetables and groundwater from organic vegetable bases, northern China. Environ. Pollut..

[22] Jin C.X., Chen Q.Y., Sun R.L., Zhou Q.X., Liu J.J. (2009). Eco-toxic effects of sulfadiazine sodium, sulfamonomethoxine sodium and enrofloxacin on wheat, Chinese cabbage and tomato. Ecotoxicology.

[23] Migliore L., Cozzolino S., Fiori M. (2003). Phytotoxicity to and uptake of enrofloxacin in crop plants. Chemosphere.

[24] Hoang T.T.T., Tu L.T.C., Le N.P., Dao Q.P., Trinh P.H. (2012). Fate of fluoroquinolone antibiotics in Vietnamese coastal wetland ecosystem. Wetl. Ecol. Manag..

[25] Tuo X.X., Gu J., Wang X.J., Sun Y.X., Duan M.L., Sun W., Yin Y.A., Guo A.Y., Zhang L. (2018). Prevalence of quinolone resistance genes, copper resistance genes, and the bacterial communities in a soil-ryegrass system co-polluted with copper and ciprofloxacin. Chemosphere.

[26] Tyrrell C., Burgess C.M., Brennan F.P., Walsh F. (2019). Antibiotic resistance in grass and soil. Access Microbiol..

[27] Mary L.G., David E.S. (2001). Fortified Foods and Phytoremediation. Two Sides of the Same Coin. Plant Physiol. (Bethesda).

[28] Yan A., Wang Y.M., Tan S.N., Yusof M.L.M., Ghosh S., Chen Z. (2020). Phytoremediation: A Promising Approach for Revegetation of Heavy Metal-Polluted Land. Front. Plant Sci..

[29] Deng S.J., Ma C.Y., Yan Y., Ye X.F., Wang G.X. (2019). Ecotoxicological Effects of Three Antibiotics on Seed Germination of Lolium perenne. Asian J. Ecotoxicol..

[30] Wei X., Wu S.C., Nie X.P., Yediler A., Wong M.H. (2009). The effects of residual tetracycline on soil enzymatic activities and plant growth. J. Environ. Sci. Health Part B.

[31] Zhao Y. (2016). Study on the Migration in Soil-Plant System and Ecological Toxicity to Plant of Two Typical Antibiotics. Master’s Thesis.

[32] Pei M., Liang Y.T., Yi L.Y., Cao S.N., Yang Z.P., Wang D.D., Zhao Y. (2017). Degradation of residual antibiotics in soils by ryegrass and its effect on microbial activity. Chin. J. Environ. Eng..

[33] Liu Q.H. (2012). The Preliminary Study about Antibiotics Residues Analysis in Herbs and Grass and Their Accumulation Pattern. Master’s Thesis.

[34] Jin L. (2014). Effects of PAHs on Endophytic Bacteria in Ryegrass and Isolation, Performance of a Phenanthrene-Degrading Endophytic Bacterium. Master’s Thesis.

[35] Ren Z.X., Xu H.H., Wang Y.W., Li Y.F., Han S., Ren J.B. (2021). Combined toxicity characteristics and regulation of residual quinolone antibiotics in water environment. Chemosphere.

[36] Mao X.W., Xue Q.G., Xia Q.F., Li D.F., Lin Z.H. (2020). Studies on genes and protein activities of extracellular superoxide dismutase homologs in Pacific oysters (*Crassostrea gigas*). J. Biol..

[37] Xu J. (2013). Research progress in superoxide dismutase and its application. Sci. Technol. Food Ind..

[38] Dong L., He Y.Z., Wang Y.L., Dong Z.Y. (2013). Research Progress on Application of Superoxide Dismutase (SOD). J. Agric. Sci. Technol..

[39] Deng F.F., Yang S.L., Gong M. (2015). Regulation of Cell Signaling Molecules on Proline Metabolism in Plants under Abiotic Stress. Plant Physiol. J..

[40] Zeng Y., Yan L., Liu Y.L., Zeng Z.J., Jiang C.C. (2020). Effects of Exogenous Proline on the Growth, Physiological Characteristics, and Proline Metabolism of Cotton Seedlings under Boron Deficiency Stress. Cotton Sci..

[41] Knapp S., Kardinahl S., Hellgren N., Tibbelin G., Schäfer G., Ladenstein R. (1999). Refined crystal structure of a superoxide dismutase from the hyperthermophilic archaeon Sulfolobus acidocaldarius at 2.2 A resolution. J. Mol. Biol..

[42] Klychnikov O.I., Shamorkina T.M., Weeks S.D., Van-Leeuwen H.C., Corver J., Drijfhout J.W., Van-Veelen P.A., Sluchanko N.N., Strelkov S.V., Hensbergen P.J. (2018). Discovery of a new Pro-Pro endopeptidase, PPEP-2, provides mechanistic insights into the differences in substrate specificity within the PPEP family. J. Biol. Chem..

[43] Ren Z.X., Wang Y., Xu H.H., Li Y.F., Han S. (2019). Fuzzy Comprehensive Evaluation Assistant 3D-QSAR of Environmentally Friendly FQs to Reduce ADRs. Int. J. Environ. Res. Public Health.

[44] Hou Y.L., Zhao Y.Y., Li Y. (2020). Environmentally Friendly Fluoroquinolone Derivatives with Lower Plasma Protein Binding Rate Designed Using 3D-QSAR, Molecular Docking and Molecular Dynamics Simulation. Int. J. Environ. Res. Public Health.

[45] Gu W.W., Li Q., Li Y. (2020). Law and mechanism analysis of biodegradability of polychlorinated naphthalenes based on principal component analysis, QSAR models, molecular docking and molecular dynamics simulation. Chemosphere.

[46] Qu R.J., Liu H.X., Feng M.B., Yang X., Wang Z.Y. (2012). Investigation on Intramolecular Hydrogen Bond and Some Thermodynamic Properties of Polyhydroxylated Anthraquinones. J. Chem. Eng. Data.

[47] Zeng X.L., Qu R.J., Feng M.B., Chen J., Wang L.S., Wang Z.Y. (2016). Photodegradation of Polyfluorinated Dibenzo-p-Dioxins in Organic Solvents: Experimental and Theoretical Studies. Environ. Sci. Technol..

[48] Gu W.W., Zhao Y.Y., Li Q., Li Y. (2019). Environmentally friendly polychlorinated naphthalenes (PCNs) derivatives designed using 3D-QSAR and screened using molecular docking, density functional theory and health-based risk assessment. J. Hazard. Mater..

[49] Könemann H. (1981). Fish toxicity tests with mixtures of more than two chemicals: A proposal for a quantitative approach and experimental results. Toxicology.

[50] Wang H.Q., Dong Y.Y., Wang L.W., Gao W.Q., Zou X.J. (2017). The Toxicity of Four Quinolones to Photobacterium phosphoreum. Asian J. Ecotoxicol..

[51] Yang L.Z., Liu M. (2019). 3D-QSAR Model of Polybrominated Biphenyls Tri-effect Modified by Standard Deviation Standardization Method and Its Application in Environmental Friendly Molecular Modification. Chem. J. Chin. Univ..

[52] Wang X.L., Gu W.W., Guo E.M., Cui C.Y., Li Y. (2017). Assessment of long-range transport potential of polychlorinated Naphthalenes based on three-dimensional QSAR models. Environ. Sci. Pollut. Res..

[53] Gu W.W., Chen Y., Li Y. (2017). Attenuation of the Atmospheric Migration Ability of Polychlorinated Naphthalenes (PCN-2) Based on Three-dimensional QSAR Models with Full Factor Experimental Design. Bull. Environ. Contam. Toxicol..

[54] Li X.L., Ye L., Shi W., Liu H.L., Liu C.S., Qian X.P., Zhu Y.L., Yu H.X. (2013). In silico study on hydroxylated polychlorinated biphenyls as androgen receptor antagonists. Ecotoxicol. Environ. Saf..

[55] Balasubramaniyan S., Irfan N., Umamaheswari A., Puratchikody A. (2018). Design and virtual screening of novel fluoroquinolone analogs as effective mutant DNA GyrA inhibitors against urinary tract infection-causing fluoroquinolone resistant Escherichia coli. RSC Adv..

[56] Yang L.Z., Liu M. (2020). A Double-Activity (Green Algae Toxicity and Bacterial Genotoxicity) 3D-QSAR Model Based on the Comprehensive Index Method and Its Application in Fluoroquinolones’ Modification. Int. J. Environ. Res. Public Health.

[57] Wu Z.G. (2014). Study on Ecological Toxicity Effects of Typical Quinolone Antibiotics in Water. Master’s Thesis.

[58] Chen L.F. (2010). The Toxicity Effect of Three Quinolone Antibiotics on Scenedesmus Obliquus. Master’s Thesis.

[59] Ebert I., Bachmann J., Kühnen U., Küster A., Kussatz C., Maletzki D., Schlüter C. (2011). Toxicity of the fluoroquinolone antibiotics enrofloxacin and ciprofloxacin to photoautotrophic aquatic organisms. Environ. Toxicol. Chem..

[60] Domagala J.M. (1994). Structure-activity and structure-side-effect relationships for the quinolone antibacterials. J. Antimicrob. Chemother..

[61] Vugia D., Cronquist A., Hadler J., Tobin-D’Angelo M., Blythe D., Smith K., Thornton K., Morse D., Cieslak P., Jones T. (2005). Preliminary FoodNetData on the Incidence of Infection with Pathogens Transmitted Commonly Through Food-10 Sites, United States, 2004. Mmwr. Morb. Mortal. Wkly. Rep..

[62] Zhao X.H., Wang X.L., Li Y. (2019). Combined HQSAR method and molecular docking study on genotoxicity mechanism of quinolones with higher genotoxicity. Environ. Sci. Pollut. Res. Int..

[63] Mao L. (2015). Common Antibiotic Genotoxicity of Veterinary Drugs on Crops. Master’s Thesis.

[64] Shi Y., Ding W. (2016). Study on the combined toxicity of binary quinolones to Vibrio Qinghaiensissp- Q67. Shaanxi J. Agric. Sci..

[65] Yang L.H., Ying G.G., Su H.C., Stauber J.L., Adams M.S., Binet M.T. (2008). Growth-inhibiting effects of 12 antibacterial agents and their mixtures on the freshwater microalga *Pseudokirchneriella subcapitata*. Environ. Toxicol. Chem..

[66] E X.-D. (2014). Study on the Salt Resistance of Several Kinds of *Lolium perenne*. Heilongjiang Agric. Sci..

[67] Zhao F.K., Chen L.D., Yang L., Fang L., Sun L., Li S.J. (2017). Composition and Distribution of Antibiotics in Soils with Different Land Use Types in a Typical Peri-urban Area of the Yangtze River Delta. Environ. Sci..

[68] Zhang X.G., Ning G.H., Liu S.Q., Zhang T.Z., Wang Y.Q., Mo C.H. (2011). Study on Eco-environment Risk Assessment of Antibiotics Concentrations in Soil and Environment in the Grapes Region of Zhangjiakou. Acta Agric. Boreali Sin..

